# HPV Infection Prevalence, Vaccination-Related Knowledge, Attitudes, and Barriers Among Women Aged 30–64 in Shenzhen, China: A Cross-Sectional Study

**DOI:** 10.3390/vaccines13060561

**Published:** 2025-05-25

**Authors:** Zhongai Ouyang, Minting Zhu, Zhijian Chen, Weigui Ni, Lijuan Lai, Bingyi Lin, Long Jiang, Yi Jing, Jingjie Fan

**Affiliations:** 1 Department of Preventive Healthcare, Shenzhen Maternity and Child Healthcare Hospital, Southern Medical University, Shenzhen 518028, China; ouyangzhongai@szmch.net.cn (Z.O.); zhuminting@szmch.net.cn (M.Z.); chenzhijian@szmch.net.cn (Z.C.); niweigui@szmch.net.cn (W.N.); lailijuan@szmch.net.cn (L.L.); linbingyi@szmch.net.cn (B.L.); jianglong@szmch.net.cn (L.J.); jy@szmch.net.cn (Y.J.); 2 School of Public Health, Southern Medical University, Guangzhou 510515, China; 3 Faculty of Medicine, Macau University of Science and Technology, Avenida Wai Long, Taipa, Macau 999078, China

**Keywords:** HPV prevalence, HPV vaccination uptake, willingness to receive HPV vaccine, related factors

## Abstract

**Background**: the distribution of human papillomavirus (HPV) infection, vaccination rates, and awareness levels varies across China. **Methods**: this study examined HPV infection prevalence, vaccine uptake, and barriers among 2440 women aged 30–64 in Shenzhen, China, using partial least squares structural equation modeling (PLS-SEM) to analyze associated factors. **Results**: The overall HPV prevalence was 14.2% (347/2440), with HPV52 being the most common type, followed by HPV58 and HPV53. Factors significantly associated with HPV infection included more sexual partners, genital tract infections, manual labor, and single marital status (*p* < 0.05), whereas higher education demonstrated a protective association (*p* < 0.05). The HPV vaccination rate was 41.8% in ages 30–45. There were direct effect indicators of younger age, fewer pregnancies, and premenopausal status (*p* < 0.05) on HPV vaccine uptake, whereas inversely associated factors included divorce/widowed, lower household income, irregular menstruation, more deliveries, no contraception, and lack of HPV knowledge. Among 828 unvaccinated individuals, 47.9% of those aged 46–64 were willing if the age restrictions were expanded, with the main barrier being a lack of vaccine knowledge (40.7%). Willingness was significantly associated with younger age and healthcare occupation (*p* < 0.05), but negatively with eastern Shenzhen residence, lower household income, no HPV disease awareness, abnormal leucorrhea, lack of HPV knowledge, and belief against post-vaccination screening (*p* < 0.05). **Conclusions**: Socioeconomic disparities in HPV infection and vaccination rates in Shenzhen highlight intervention priorities. The impact of HPV knowledge underscores the need for effective health communication. The vaccination willingness and infection status among women aged 45+ provide supporting evidence for expanding HPV vaccination to older age groups.

## 1. Introduction

Cervical cancer is the fourth most common cancer in women globally [1]. In China, it remains a major threat to women’s health, with approximately 150,700 new cases and 55,700 deaths reported in 2022 by the International Agency for Research on Cancer (IARC) [2]. Over the past two decades, cervical cancer incidence and mortality rates have steadily risen [3]. Extensive research has demonstrated that nearly all cases of cervical cancer are caused by persistent infection with high-risk human papillomavirus (HR-HPV) [4]. As there are no effective methods to completely clean a persistent HR-HPV infection, cervical cancer prevention primarily relies on HPV vaccination and regular screening [5,6]. HPV vaccination is pivotal to the WHO’s cervical cancer elimination strategy [7]. China has approved the following three vaccines for females: bivalent (HPV16/18; ages 9–45, 2016), quadrivalent (HPV16/18/6/11; ages 9–45, 2017), and nine-valent (HPV16/18/6/11/31/33/45/52/58; ages 16–26 to 9–45 after 2022 expansion). In 2025, the following approvals were extended to males: quadrivalent (ages 9–26) and nine-valent (ages 16–26).

Recent studies have consistently reported insufficient HPV vaccine awareness, poor knowledge regarding vaccination benefits, and low uptake rates throughout mainland China [8]. Socioeconomic disparities in HPV-related knowledge and vaccination uptake are evident in China [9,10]. Given the persistently low HPV vaccination rate nationwide, increasing coverage is crucial to reducing cervical cancer incidence and mortality [11]. Knowledge about HPV and its role in cancer development was very low among women recruited from Yunnan Province in southwestern China [10]. In Gansu Province, located in northwestern China, the first-dose HPV vaccination rate among women aged 9–45 years from 2018 to 2021 was only 2.02%, with limited awareness and low vaccine trust identified as key barriers [12,13]. Eligible female populations in Shenzhen, representing China’s developed coastal cities, exhibited substantially greater awareness of cervical cancer prevention and HPV vaccination benefits than national averages [14]. Tobit model analysis suggests that improving public HPV vaccine knowledge could significantly enhance coverage [15].

Cervical cancer incidence rates are higher in Asian women compared with most European countries [16]. South Korea and Japan recommend regular HPV screening from 20 years old, effectively reducing incidence [17,18]. However, studies suggest that HPV testing for women under 30 may increase overdiagnosis, overtreatment, and costs [19]. A study in the United States found that initiating low-cost HPV testing at age 30, with a five-year interval, achieves a reasonable balance between benefits, harms, and costs [20]. In the guideline for screening and treatment of cervical pre-cancer lesions for cervical cancer prevention (second edition) released by WHO in July 2021, it is strongly recommended that the general population begin regular screening from the age of 30 [21]. In China, the recommended upper age limit for screening has been 64 since 2015. The 2017 comprehensive guidelines for cervical cancer prevention, developed by Chinese gynecological experts, further suggest that women over 65 may discontinue screening if they have had adequate screening in the past 10 years and no history of cervical intraepithelial neoplasia (CIN) [22].

Most existing studies rely on hospital-based data, which may compromise population representativeness and the accuracy of infection rate estimates. To address the heterogeneity in HPV testing practices across Shenzhen (assays ranging from 2 to 23 subtypes with variable quality), we conducted the city’s first population-based survey using a standardized 23-genotype assay from a single manufacturer, ensuring methodological consistency and optimal detection sensitivity. Considering both international screening age recommendations and local budget constraints, we determined that adopting a standardized 23-genotype HPV testing protocol for women aged 30–64 in Shenzhen offers the most cost-effective approach. This strategy enabled the comprehensive assessment of HPV prevalence, identification of risk factors, and evaluation of vaccination barriers among this key demographic group. We believe the methodological framework and findings may offer valuable insights for other similar urban centers in China, particularly with comparable demographic characteristics and healthcare infrastructures.

The purpose of this study was to explore HPV infection rates and associated factors among healthy women aged 30–64 in Shenzhen. The second objective was to analyze factors influencing HPV vaccination uptake and willingness to vaccinate among unvaccinated women.

## 2. Materials and Methods

### 2.1. Study Design

Shenzhen, one of China’s most developed cities, has a population of 17.56 million (44.96% female, 2020 National Census) [23] and geographically convenient access to Hong Kong and Macao. Prior to HPV vaccine approval in mainland China, awareness of its benefits was already widespread in Shenzhen through commercial advertising and word-of-mouth, leading to higher acceptance and cross-border vaccination uptake [24]. Although HPV vaccination remains self-funded nationally, Guangdong Province (including Shenzhen) launched a free bivalent HPV vaccination program for first year junior high school girls in September 2022.

From September 2024 to March 2025, we conducted a cross-sectional study (File S1) across ten districts in Shenzhen, representing eastern (Longgang, Pingshan, Dapeng), western (Nanshan, Baoan, Guangming), and central (Futian, Luohu, Yantian, Longhua) regions. Participants completed structured questionnaires (File S2) and underwent HPV testing using a standardized 23-subtype assay from a single manufacturer.

The study was approved by the Institutional Ethics Committees of the Chinese Academy of Medical Sciences & Peking Union Medical College (CAMS&PUMC-IEC-2024-003) and Shenzhen Maternity and Child Healthcare Hospital (SFYLS [2025]013). Due to ethical approval timelines, the recruitment of the 45–64 age group was conducted from September 2024 to March 2025, while the 30–44 age group was recruited in March 2025 as soon as possible after obtaining ethical approval. All participants provided written informed consent. This study adhered to the STROBE guidelines for observational studies [25] and incorporated methodological standards for survey research, including response rate calculation [26,27].

### 2.2. Criteria for Participants

**Figure 1:**
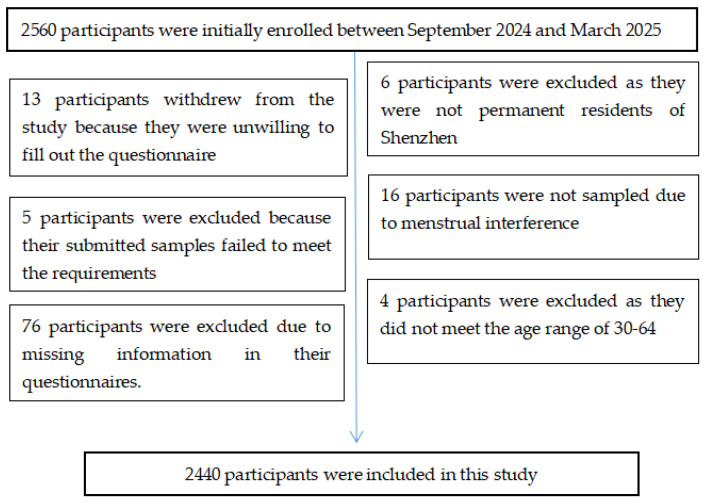
Inclusion process of study subjects.

Participants were randomly sampled from ten districts in Shenzhen using a stratified random sampling method. The sample size was calculated based on Shenzhen’s female demographic data (China’s Seventh National Population Census, 2020) [23], stratified by district and age group. Using PASS 11.0 with an assumed HPV prevalence of 14.86% (derived from recent urban studies) [28], we determined a minimum required sample of 2305. To account for potential non-response, we increased this by 10%, resulting in a final recruit target sample of 2536 participants. Figure 1 shows the sample exclusion process and inclusion results, with a final sample size of 2440 cases.

Before the survey began, all participants were informed of the purpose and procedures of this study, and informed consent was obtained. This study enrolled Shenzhen female residents who met the following criteria: (1) aged 30–64; (2) history of sexual activity at any age; (2) voluntarily consenting to HPV testing and questionnaire completion; (3) abstinence from sexual intercourse and vaginal medications for ≥48 h prior to sampling. Exclusion criteria were as follows: (1) active infection or autoimmune disease; (2) current pregnancy; (3) history of cervical surgery or hysterectomy (with cervical removal); (4) cervical cancer patients; and (5) history of immunosuppressive therapy. We employed the aforementioned methods to minimize potential biases in this study.

### 2.3. Definition of Variables

The self-administered questionnaire systematically evaluated several key domains: demographic characteristics (including age, education level, marital status, and household income), obstetric and gynecological history (parity and pregnancy), sexual behavior (age at first intercourse and lifetime number of sexual partners), HPV-related knowledge, vaccine uptake status, and willingness for future vaccination.

#### 2.3.1. HPV Knowledge and HPV Vaccination

Participants’ knowledge was assessed using an 8-item scale covering HPV, cervical cancer, screening, and vaccination. Responses were scored as 1 (correct) or 0 (incorrect/“don’t know”), with total scores ranging from 0 to 8. A median score of 5 or above indicated a high knowledge level. The reliability of knowledge items was evaluated by assessing the internal consistency of the items representing the knowledge score. The 8 knowledge items in the study sample had a reliability (Kuder–Richardson 20) of 0.814.

#### 2.3.2. Practice and Willingness to Receive HPV Vaccination

Participants reported their HPV vaccination status. Unvaccinated individuals were asked about their willingness to receive the vaccine, with those over 45 questioned on future availability. Responses were “yes” or “no”. The questionnaire was reviewed by experts and pilot-tested on 10 individuals, who were excluded from the final study.

#### 2.3.3. HPV Genotyping

HPV DNA typing was performed using a genotyping kit (Kaipu Biotechnology, Chaozhou, China), which detected 23 common types (14 high-risk, 3 middle-risk, and 6 low-risk) through DNA amplification with HPV L1 consensus PCR primers and flow-through hybridization. All procedures followed the manufacturer’s instructions [29].

#### 2.3.4. Histopathological Diagnosis

Among the 347 participants infected with HPV, 128 who consented had TCT for further cervical testing, with results interpreted using the Bethesda 2014 criteria [30]. TCT pathological results were categorized as follows: (a) normal cervix; (b) atypical squamous cells (ASC-US/-H); (c) low-grade squamous intraepithelial lesion (LSIL, CIN I); (d) high-grade squamous intraepithelial lesion (HSIL, CIN II/III).

### 2.4. Statistical Analysis

Data were independently entered using EpiData and analyzed with SPSS 27.0 (IBM, USA). Quantitative variables were presented as median (interquartile range, IQR). Univariate analyses using Chi-square or Fisher’s exact tests identified potential factors associated with HPV status, vaccine uptake, and vaccination willingness. Given the non-normal distribution of our data and the presence of multicollinearity among variables, we employed PLS-SEM as our primary analytical approach. Significant variables (*p* < 0.05) from univariate analyses were subsequently analyzed by PLS-SEM using SmartPLS 4.0 (SmartPLS GmbH, Hamburg, Germany), with bootstrapping (5000 resamples) to assess model robustness.

## 3. Results

### 3.1. HPV Infection and Type Distribution

**Figure 2:**
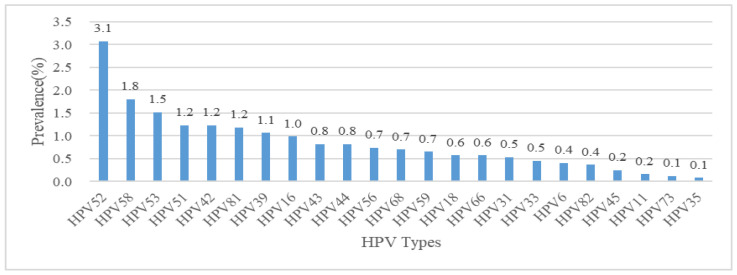
The distribution of HPV genotypes in Shenzhen, China.

**Figure 3:**
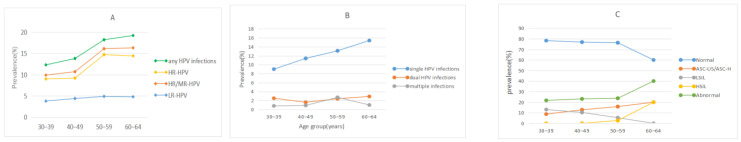
The prevalence of HPV infection by age groups. (**A**) High-risk, high-risk/middle-risk, and low-risk HPV infection and overall HPV infection; (**B**) single, double, and multiple infection; (**C**) TBS diagnostic results.

Among 2440 females (median age 40, IQR 35–49), the overall HPV prevalence was 14.2%. The most prevalent types were HPV52 (3.1%, 75/2440), HPV58 (1.8%, 44/2440), and HPV53 (1.5%, 37/2440) (Figure 2). HR-HPV, HR/middle-risk HPV, and LR-HPV prevalence rates were 10.5% (256/2440), 11.6% (283/2440), and 4.2% (103/2440), respectively. HPV and HR/middle-risk HPV prevalence increased with age, peaking at 19.2% (20/104) and 16.3% (17/104) in the 60–64 age group, while HR-HPV and LR-HPV peaked at 14.7% (72/490) and 4.9% (24/490) in the 50–59 age group. The Cochran–Armitage trend test showed a significant linear increase in HPV, HR-HPV, and HR/middle-risk HPV prevalence with age (*p* < 0.001) (Figure 3A, Table 1).

Single, dual, and multiple HPV infections were detected in 10.8% (236/2440), 2.3% (55/2440), and 1.2% (29/2440) of participants, respectively. The Cochran–Armitage trend test revealed a significant linear increase in single and multiple HPV infection rates with age (*p* < 0.05) (Figure 3B, Table 1).

Based on the Bethesda system (TBS), among the 128 participants with HPV infection, cytological results were categorized as follows: normal (n = 98), ASC (ASC-US, n = 12; ASC-H, n = 4), LSIL (n = 12), and HSIL (n = 2). Abnormal TBS results peaked in the 60–64 age group (40.0%, 2/5) and were lowest in the 30–39 group (21.7%, 10/46). ASC and HSIL prevalence peaked in the 60–64 group (20.0%, 2/5), while LSIL was highest in the 30–39 group (13.0%, 6/46). The Cochran–Armitage trend test showed a significant linear increase in HSIL prevalence with age (*p* < 0.05) (Figure 3C, Table 1).

### 3.2. HPV Infection Status

Among 2440 participants, 347 (14.2%) had HPV infection, and 2093 (85.8%) showed no evidence of infection. Univariate analysis showed that the absence of HPV infection was significantly associated with younger age, Han ethnicity, being married, healthcare occupation, higher education (participants and spouses), higher household income, first intercourse after age 20, fewer sexual partners, lower sexual frequency, non-menopausal status, contraceptive use, absence of genital tract infections, HPV awareness, knowledge of HPV-related diseases, and higher HPV knowledge scores (Table 2).

**Figure 4:**
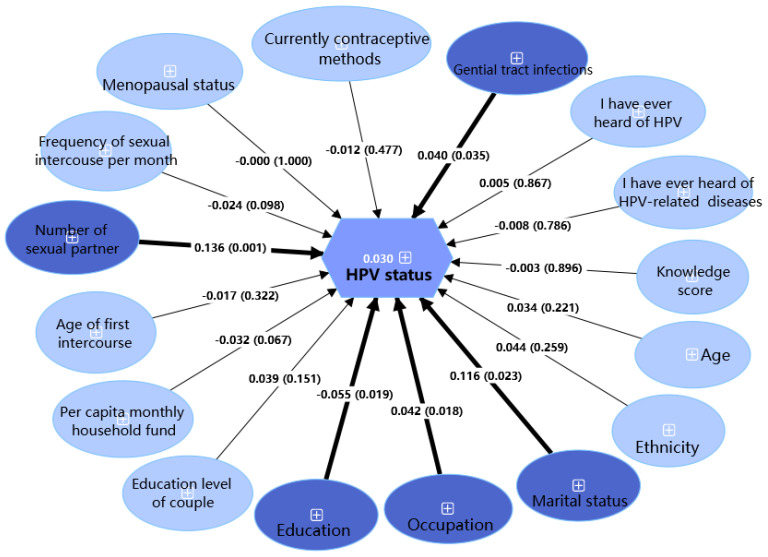
Partial least squares structural equation model of factors predicting HPV infection status among healthy female residents in Shenzhen (N = 2440). Dark blue: statistically significant factors in PLS-SEM (*p* < 0.05); light blue: non-significant factors in PLS-SEM (*p* ≥ 0.05); thick lines: significant paths (*p* < 0.05); thin lines: non-significant paths (*p* ≥ 0.05).

Figure 4, generated using PLS-SEM, identifies factors influencing HPV status. The model shows a significant association between HPV status and having more sexual partners (β = 0.136, *p* < 0.05), genital tract infections (β = 0.040, *p* < 0.05), manual labor (β = 0.042, *p* < 0.05), and being single (β = 0.116, *p* < 0.05). Higher education was protective (β = −0.055, *p* < 0.05). The model explains 3.0% of HPV status variance (adjusted R^2^ = 0.030).

### 3.3. HPV Vaccine Uptake

Among 2440 participants, 704 (28.9%) reported receiving the HPV vaccine, with a higher uptake rate of 41.8% among those aged 30–45. Univariate analyses indicated significant associations between most variables and HPV vaccine uptake (Table 3).

**Figure 5:**
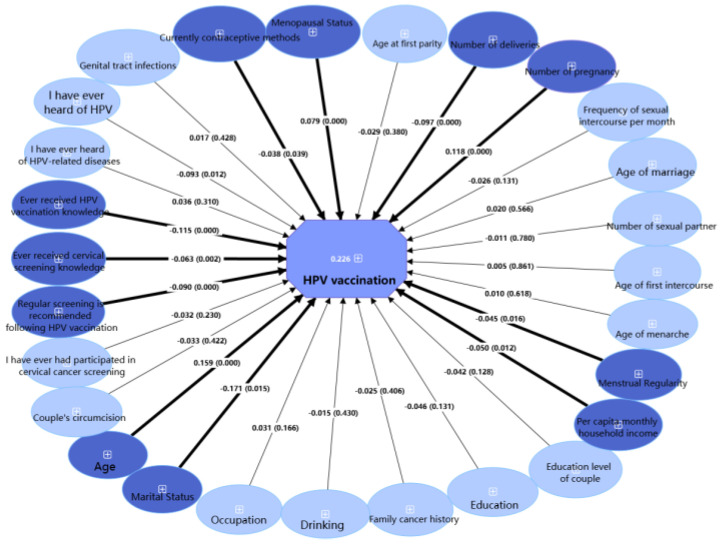
Partial least squares structural equation model of factors predicting HPV vaccination uptake among healthy female residents in Shenzhen (N = 2440). Dark blue: statistically significant factors in PLS-SEM (*p* < 0.05); light blue: non-significant factors in PLS-SEM (*p* ≥ 0.05); thick lines: significant paths (*p* < 0.05); thin lines: non-significant paths (*p* ≥ 0.05).

Figure 5, analyzed using PLS-SEM, identified factors influencing HPV vaccine uptake. Younger age (β = 0.159, *p* < 0.05), fewer pregnancies (β = 0.118, *p* < 0.05), and non-menopausal status (β = 0.079, *p* < 0.05) were associated with higher HPV vaccine uptake, whereas divorced/widowed status (β = −0.171, *p* < 0.05), lower household income (β = −0.050, *p* < 0.05), irregular menstruation (β = −0.045, *p* < 0.05), more deliveries (β = −0.097, *p* < 0.05), non-use of contraception (β = −0.038, *p* < 0.05), lack of HPV awareness (β = −0.093, *p* < 0.05), non-receipt of vaccination information (β = −0.115, *p* < 0.05), non-receipt of screening information (β = −0.063, *p* < 0.05), and lack of understanding of post-vaccination screening recommendations (β = −0.090, *p* < 0.05) was associated with reduced vaccine uptake. The model explained 22.6% of vaccine uptake variance (adjusted R^2^ = 0.226).

### 3.4. Willingness to Receive the HPV Vaccination

**Figure 6:**
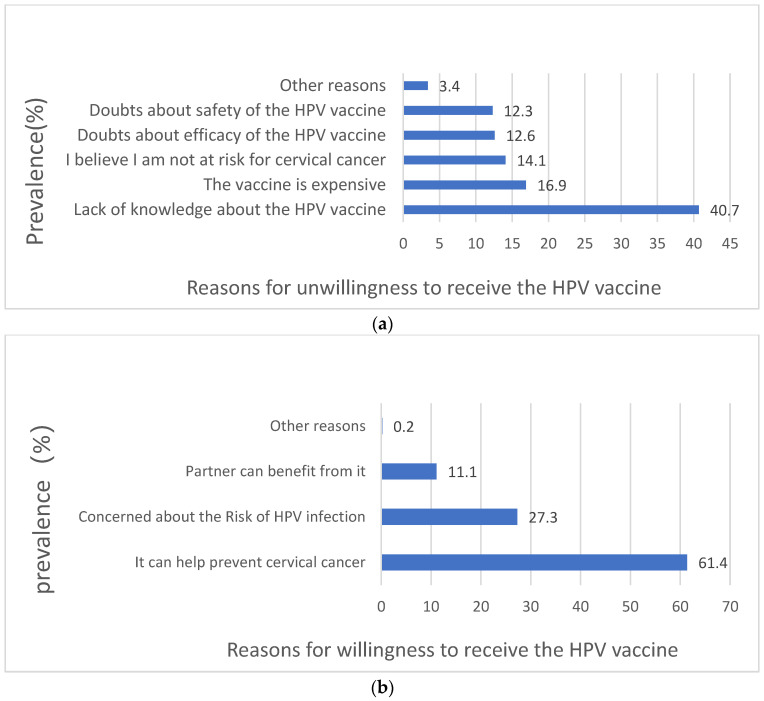
Reasons for willingness and unwillingness to receive the HPV vaccine among unvaccinated individuals. (**a**) Reasons for unwillingness to receive the HPV vaccine; (**b**) reasons for willingness to receive the HPV vaccine.

Among 1736 unvaccinated participants, 60.6% (n = 1052) expressed willingness to receive the HPV vaccine, with 47.9% of those aged 46–64 willing to vaccinate. Univariate analyses revealed significant associations between most variables and vaccination willingness (Table 4). The primary reasons for unwillingness included lack of vaccine knowledge (40.7%), high cost (16.9%), and perceived low cervical cancer risk (14.1%). Willingness was primarily driven by belief in the vaccine’s efficacy in preventing cervical cancer (61.4%), concern about HPV infection (27.3%), and partner benefits (11.1%) (Figure 6a,b).

**Figure 7:**
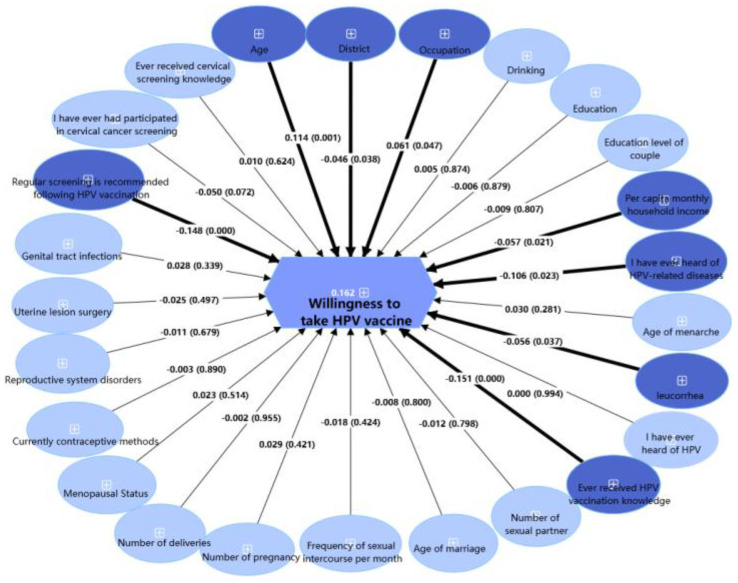
Partial least squares structural equation model of factors predicting willingness to receive HPV vaccination among healthy female residents in Shenzhen (N = 1736). Dark blue: statistically significant factors in PLS-SEM (*p* < 0.05); light blue: non-significant factors in PLS-SEM (*p* ≥ 0.05); thick lines: significant paths (*p* < 0.05); thin lines: non-significant paths (*p* ≥ 0.05).

Figure 7, analyzed using PLS-SEM, identified factors influencing HPV vaccine willingness. Higher HPV vaccine willingness was significantly associated with younger age (β = 0.114, *p* < 0.05) and medical profession status (β = 0.061, *p* < 0.05). Significantly lower HPV vaccine willingness was associated with residence in eastern Shenzhen (β = −0.046, *p* < 0.05), lower household income (β = −0.057, *p* < 0.05), no awareness of HPV-related diseases (β = −0.106, *p* < 0.001), abnormal leucorrhea (β = −0.056, *p* < 0.05), lack of HPV vaccination knowledge (β = −0.151, *p* < 0.05), and opposition to post-vaccination screening (β = −0.148, *p* < 0.001). The model explained 16.2% of willingness variance (adjusted R^2^ = 0.162).

## 4. Discussion

Our standardized testing yielded reliable and homogeneous results, with an HPV infection rate of 14.2%, comparable to Guangzhou (16.01%) [31], lower than Shanghai (18.81%) [32] but higher than Beijing (11.9%) [33]. The most prevalent subtypes were HPV52, HPV58, and HPV53, consistent with findings from East Asia [34]. Our study found HPV infection rates increased with age, potentially due to reduced hormone levels and weakened immunity in peri- and post-menopausal women [35]. Among 128 participants infected with HPV identified via TCT screening, 30 cases (23.4%) showed abnormal cytology results. Within these abnormal cases, HPV52 demonstrated the highest prevalence (36.7%, 11/30), whereas HPV16 and 18 exhibited significantly lower detection rates (3.4% [1/30] and 6.7% [2/30]). This genotype distribution contrasts markedly with prior Shenzhen research identifying HPV52/16/18 as predominant in histologically confirmed cervical lesions [36], suggesting our TCT results may not fully represent HPV-infected populations, limiting genotype severity conclusions. PLS-SEM results showed HPV-infected status was significantly associated with more sexual partners, genital tract infections, manual labor, being single, and lower education, consistent with a Brazilian study identifying the number of sexual partners as a key determinant [37]. Increased genital HPV prevalence may result from viral entry through skin or mucosal injuries, facilitated by physical contact or trauma during intercourse [38].

Since HPV vaccines were first approved in mainland China in 2016, vaccination rates among participants aged 30–45 reached 41.8% and 28.8% for those aged 30–64 in this study, a nearly 14-fold increase from the 3% rate in 2019 [8]. This study found that the majority of participants were aware of HPV and the HPV vaccine, with a moderate-to-high level of knowledge (5.2/8). Furthermore, 80% of participants reported awareness of the HPV vaccine, representing a 2.5-fold increase compared with the 34% awareness rate reported in 2019 [8]. Identifying the factors that influence HPV vaccination among women in Shenzhen is crucial for developing targeted policies to improve vaccination rates. Our study revealed that key factors associated with vaccine uptake included lower monthly household income per capita, pregnancy and delivery history, and awareness and knowledge of HPV. This aligns with findings from a study in South Korea, where low income and limited awareness were associated with a lower willingness to receive the HPV vaccine [39]. Moreover, our findings reveal that vaccine hesitancy is associated with limited HPV knowledge and misconceptions about cervical cancer susceptibility. This underscores the critical need for public health education to communicate the well-established efficacy of HPV vaccination in preventing CIN2+ and cervical cancer, as demonstrated by numerous studies [40,41,42,43]. Importantly, studies have demonstrated that women with pre-existing HR-HPV infections can still benefit from vaccination [44].

This study revealed that 60.6% of 1736 unvaccinated individuals were willing to self-fund HPV vaccination, with 47.9% of 828 unvaccinated individuals aged 46–64 expressing willingness to self-fund vaccination if age restrictions were expanded in China. Notably, this older cohort (45+) demonstrates elevated HPV infection rates like this research [45], yet remains understudied regarding vaccination attitudes. These findings provide the empirical evidence supporting age-expansion policies for HPV vaccination in China, addressing a critical gap in preventive strategies for this high-risk population. In total, 61.2% of participants had undergone cervical cancer screening, including free national screening programs and self-funded screenings. Among those aged 35–64, 63.6% had participated in screening, approaching the WHO’s target coverage rate of 70% [7]. Key vaccination barriers included limited HPV awareness and cost concerns. PLS-SEM showed lower willingness among low-income earners, manual laborers, and those with limited HPV awareness, consistent with Romanian findings [46], underscoring the need for HPV education. The current efforts in China show progress but face challenges like vaccine supply and equitable access [47]. While China has not yet included the HPV vaccine in its national immunization program due to financial constraints, some regions with sufficient resources have taken the lead. For example, Guangdong Province, where Shenzhen is located, initiated a provincial immunization program in September 2022, providing free bivalent HPV vaccinations to eligible girls in the first year of junior high school. Furthermore, with the approval of the quadrivalent and nine-valent HPV vaccine for new indication in 2025, males aged 9–26 will also be eligible for vaccination. This expansion marks a significant step forward in China’s efforts to combat cervical cancer and improve overall HPV-related disease prevention.

Furthermore, the evidence suggests that combining the HPV vaccination with quinquennial screening represents the most cost-effective strategy for cervical cancer prevention in China. Reducing the two-dose vaccine cost to under USD 50 would enhance cost-effectiveness compared with screening alone, even at low willingness-to-pay thresholds [48]. Future efforts should focus on improving screening and vaccination rates to maximize cost-effectiveness. A systematic review from Africa found educational interventions, particularly peer health education and culturally appropriate methods, effectively increase cervical cancer awareness, knowledge, and screening rates [49]. Therefore, health education on HPV and cervical cancer prevention should be implemented in communities and hospitals to improve public awareness, vaccination rates, and screening participation.

### Limitations

Our study has several important limitations. First, the implementation of stratified sampling may have been compromised by being restricted to a single center, potentially affecting the generalizability of our findings to broader populations. Second, the cross-sectional design precluded causal inference, and the lack of follow-up with participants infected with HPV prevented the assessment of persistent infection or viral clearance. Third, potential reporting bias existing as sensitive information (e.g., sexual behavior, vaccination history) relied on self-reporting. Additionally, budgetary constraints limited our study to females, excluding males who represent important transmitters and vaccination beneficiaries. Future studies should adopt multicenter longitudinal designs with expanded sampling frames to include males, enabling a more comprehensive evaluation of HPV epidemiology and prevention strategies.

## 5. Conclusions

This study found an overall HPV prevalence of 14.2% (HR-HPV: 10.5%; single-type: 10.8%), with HPV52, 58, and 53 being predominant. Infection rates increased with age, while vaccination coverage reached 41.8% in women aged 30–45. Notably, 47.9% (396/828) of unvaccinated women aged 46–64 expressed willingness to vaccinate if eligible, providing evidence to support the age expansion of HPV vaccination programs. Socioeconomic factors and HPV knowledge significantly predicted infection risk and vaccine uptake. These results highlight the imperative for targeted interventions to improve vaccine accessibility and public education, particularly among older cohorts and disadvantaged populations.

## Figures and Tables

**Table 1:** The prevalence of HPV infection and the distribution of TBS diagnostic results were analyzed across different age groups. (N = 2440 and N = 128).

HPV Genotype	N (%) (N = 2440)	30–39 (%) (n = 1142)	40–49 (%) (n = 704)	50–59 (%) (n = 490)	60–64 (%) (n = 104)	*X* ^2^	*p*	*p* ^a^
Type of infection								
HPV	347 (14.2)	141 (12.3)	97 (13.8)	89 (18.2)	20 (19.2)	11.784	<0.05	<0.001
HR-HPV	255 (10.5)	103 (9.0)	65 (9.2)	72 (14.7)	15 (14.4)	14.796	<0.05	<0.001
HR/MR-HPV	284 (11.6)	113 (9.9)	75 (10.7)	79 (16.1)	17 (16.3)	15.860	<0.05	<0.001
Lr-HPV	103 (4.2)	43 (3.8)	31 (4.4)	24 (4.9)	5 (4.8)	1.288	0.732	0.275
Number of HPV infection								
Single	263 (10.8)	103 (9.0)	80 (11.4)	64 (13.1)	16 (15.4)	8.875	<0.05	<0.05
Dual	55 (2.3)	29 (2.5)	11 (1.6)	12 (2.4)	3 (2.9)	2.222	0.528	0.880
Multiple	29 (1.2)	9 (0.8)	6 (0.9)	13 (2.7)	1 (1.0)	11.232	<0.05	<0.05
TBS diagnostic	N (%) (N = 128)	30–39 n (%) (n = 46)	40–49 n (%) (n = 39)	50–59 n (%) (n = 38)	60–64 n (%) (n = 5)	*X* ^2^	*p*	*p* ^a^
Pathological type								
Normal	98 (76.6)	36 (78.3)	30 (76.9)	29 (76.3)	3 (60.0)	0.755	0.860	0.559
Abnormal	30 (23.4)	10 (21.7)	9 (23.1)	9 (23.7)	2 (40.0)	0.755	0.860	0.559
ASC-US/ASC-H	16 (12.5)	4 (8.7)	5 (12.8)	6 (15.8)	1 (20.0)	1.249	0.741	0.268
LSIL	12 (9.4)	6 (13.0)	4 (10.3)	2 (5.3)	0 (0.0)	2.562	0.464	0.160
HSIL	2 (1.6)	0 (0.0)	0 (0.0)	1 (2.6)	1 (20.0)	6.351	0.096	<0.05

^a^: Cochran–Armitage’s *p*.

**Table 2:** Demographic characteristics and factors associated with HPV infection status among participants (N = 2440).

Univariate Analysis					
HPV State					
Demographic Variables	Overall	Positive (n = 347)	Negative (n = 2093)	χ^2^	*p*
Age group					
30–39	1142 (46.8)	141 (12.3)	1001 (87.7)	11.784	<0.05
40–49	704 (28.9)	97 (13.8)	607 (86.2)		
50–59	490 (20.1)	89 (18.2)	401 (81.8)		
60–64	104 (4.3)	20 (19.2)	84 (80.8)		
Ethnicity					
Han	2323 (95.2)	323 (13.9)	2000 (86.1)	3.988	<0.05
Other	117 (4.8)	24 (20.5)	93 (79.5)		
District *					
Eastern	707 (29.0)	101 (14.3)	606 (85.7)	1.413	0.493
Western	722 (29.6)	111 (15.4)	611 (84.6)		
Middle	1011 (41.4)	135 (13.4)	876 (86.6)		
Marital status					
Single	45 (1.8)	12 (26.7)	33 (73.3)	15.838	<0.001
Divorced/widowed	101 (4.1)	25 (24.8)	76 (75.2)		
Married	2294 (94.0)	310 (13.5)	1984 (86.5)		
Occupation					
Medical and health personnel	482 (19.8)	41 (8.5)	441 (91.5)	31.907	<0.001
Non-manual labor	841 (34.5)	101 (12.0)	740 (88.0)		
Manual labor	1117 (45.8)	205 (18.4)	912 (81.6)		
Smoking					
Never	2418 (99.1)	344 (14.2)	2074 (85.8)	0.379	0.827
Past (abstained ≥3 months)	11 (0.5)	2 (18.2)	9 (81.8)		
Current (over 6 months)	11 (0.5)	1 (9.1)	10 (90.9)		
Drinking					
Never	1636 (67.0)	217 (13.3)	1419 (86.7)	5.928	0.052
Often (<3 times/week)	795 (32.6)	127 (16.0)	668 (84.0)		
Usually (3–7 times/week)	9 (0.4)	3 (33.3)	6 (66.7)		
Family cancer history					
No	2225 (91.2)	320 (14.4)	1905 (85.6)	0.535	0.465
Yes	215 (8.8)	27 (12.6)	188 (87.4)		
Education					
Second school or below	869 (35.6)	165 (19.0)	704 (81.0)	30.501	<0.001
Senior and vocational high school	437 (17.9)	65 (14.9)	372 (85.1)		
College or above	1134 (46.5)	117 (10.3)	1017 (89.7)		
Education level of couple					
Second school or below	770 (31.6)	142 (18.4)	628 (81.6)	17.520	<0.001
Senior and vocational high school	522 (21.4)	71 (13.6)	451 (86.4)		
College or above	1148 (47.0)	134 (11.7)	1014 (88.3)		
Per capita monthly household income					
<yuan 2000	829 (34.0)	142 (17.1)	679 (83.0)	16.332	<0.001
yuan 2000–3999	592 (24.3)	94 (15.9)	505 (83.9)		
≥yuan 4000	1019 (41.8)	111 (10.9)	909 (89.1)		
**Obstetric and gynecologic** **variables**					
Menstrual regularity					
No	546 (22.4)	84 (15.4)	462 (84.6)	0.780	0.377
Yes	1894 (77.6)	263 (13.9)	1631 (86.1)		
Dysmenorrhea					
No	1711 (70.1)	244 (14.3)	1467 (85.7)	0.007	0.932
Yes	729 (29.9)	103 (14.1)	626 (85.9)		
Age of menarche					
<13	531 (21.8)	73 (13.7)	458 (86.3)	0.878	0.645
13-15	1548 (63.4)	217 (14.0)	1331 (86.0)		
>15	361 (14.8)	57 (15.8)	304 (84.2)		
Leucorrhea					
No	1831 (75.0)	250 (13.7)	1581 (86.3)	1.937	0.164
Yes	609 (25.0)	97 (15.9)	512 (84.1)		
Couple’s circumcision					
Unknown	215 (8.8)	36 (16.7)	179 (83.3)	1.606	0.448
No	1936 (79.3)	274 (14.2)	1662 (85.8)		
Yes	289 (11.8)	37 (12.8)	252 (87.2)		
Age of sexual debut					
≤20	665 (27.3)	119 (17.9)	546 (82.1)	10.112	<0.05
>20	1775 (72.7)	228 (12.8)	1547 (87.2)		
Number of sexual partners					
1	1976 (81.0)	256 (13.0)	1720 (87.0)	21.905	<0.001
2	344 (14.1)	58 (16.9)	286 (83.1)		
≥3	120 (4.9)	33 (27.5)	87 (72.5)		
Age of marriage					
≤20	299 (12.5)	52 (17.4)	247 (82.6)	3.302	0.069
>20	2097 (87.5)	283 (13.5)	1814 (86.5)		
Frequency of sexual intercourse per month					
<4	1355 (55.5)	215 (15.9)	1140 (84.1)	6.766	<0.05
≥4	1085 (44.5)	132 (12.2)	953 (87.8)		
Number of pregnancies					
<2	534 (21.9)	66 (12.4)	468 (87.6)	1.942	0.163
≥2	1906 (78.1)	281 (14.7)	1625 (85.3)		
Number of deliveries					
<2	929 (38.1)	138 (14.9)	791 (85.1)	0.493	0.482
≥2	1511 (61.9)	209 (13.8)	1302 (86.2)		
Age at first parity					
≤20	170 (7.3)	30 (17.6)	140 (82.4)	1.932	0.165
>20	2160 (92.7)	298 (13.8)	1863 (86.2)		
Menopausal status					
No	1901 (77.9)	251 (13.2)	1650 (86.8)	7.307	<0.05
Yes	539 (22.1)	96 (17.8)	443 (82.2)		
**Prior experience and medical history**					
Current contraceptive methods (like condom, contraceptives pills, sterilization, IUD)					
No	854 (35.0)	142 (16.6)	712 (83.4)	6.236	<0.05
Yes	1586 (65.0)	205 (12.9)	1381 (87.1)		
Ever had reproductive system disorders (like uterine myomas, cervical polyps, cervicitis, endometriosis)					
No	1722 (70.6)	236 (13.7)	1486 (86.3)	1.279	0.258
Yes	718 (29.4)	111 (15.5)	607 (84.5)		
Ever had uterine surgery (like hysterectomy with cervical preservation, cervical conization, cervical cerclage, polypectomy, LEEP)					
No	2118 (86.8)	300 (14.2)	1818 (85.8)	0.043	0.836
Yes	322 (13.2)	47 (14.6)	275 (85.4)		
Genital tract infections (like gonococcal, chlamydia trachomatis, mycoplasma, trichomoniasis)					
No	1952 (80.0)	262 (13.4)	1690 (86.6)	5.110	<0.05
Yes	488 (20.0)	85 (17.4)	403 (82.6)		
**HPV knowledge**					
I have ever heard of HPV					
No	684 (28.0)	114 (16.7)	570 (83.3)	4.659	<0.05
Yes	1756 (72.0)	233 (13.3)	1523 (86.7)		
I have ever heard of HPV-related diseases, such as genital warts and cervical cancer					
No	865 (35.5)	140 (16.2)	725 (83.8)	4.236	<0.05
Yes	1575 (64.5)	207 (13.1)	1368 (86.9)		
I have ever heard of HPV vaccine					
No	487 (20.0)	79 (16.2)	408 (83.8)	1.996	0.158
Yes	1953 (80.0)	268 (13.7)	1685 (86.3)		
I have ever received HPV vaccination					
No	1736 (71.1)	253 (14.6)	1483 (85.4)	0.613	0.434
Yes	704 (28.9)	94 (13.4)	610 (86.6)		
Ever received HPV vaccination knowledge					
No	489 (20.0)	79 (16.2)	410 (83.8)	1.875	0.171
Yes	1951 (80.0)	268 (13.7)	1683 (86.3)		
Ever received cervical screening knowledge					
No	947 (38.8)	142 (15.0)	805 (85.0)	0.759	0.384
Yes	1493 (61.2)	205 (13.7)	1288 (86.3)		
Regular screening is recommended following HPV vaccination					
No	191 (7.8)	36 (18.8)	155 (81.2)	4.006	0.135
Unknown	526 (21.6)	77 (14.6)	449 (85.4)		
Yes	1723 (70.6)	234 (13.6)	1489 (86.4)		
I have ever participated in cervical cancer screening					
No	947 (38.8)	142 (15.0)	805 (85.0)	0.765	0.682
National (free)	834 (34.2)	114 (13.7)	720 (86.3)		
Self-paid	659 (27.0)	91 (13.8)	568 (86.2)		
Score					
<5	771 (31.6)	129 (16.7)	642 (83.3)	5.822	<0.05
≥5	1669 (68.4)	218 (13.1)	1451 (86.9)		

* District: eastern (Longgang, Pingshan, Dapeng), western (Nanshan, Baoan, Guangming), and central (Futian, Luohu, Yantian, Longhua).

**Table 3:** Demographic characteristics and factors associated with HPV vaccine uptake among participants (N = 2440).

Univariate Analysis					
HPV Vaccination				χ^2^	*p*
Demographic Variables	Overall	Yes (n = 704)	No (n = 1736)		
Age Group					
30–39	1142 (46.8)	497 (43.5)	645 (56.5)	328.545	<0.001
40–49	704 (28.9)	195 (27.7)	509 (72.3)		
50–59	490 (20.1)	11 (2.2)	479 (97.8)		
60–64	104 (4.3)	1 (1.0)	103 (99.0)		
Ethnicity					
Han	2323 (95.2)	671 (28.9)	1652 (71.1)	0.025	0.874
Other	117 (4.8)	33 (28.2)	84 (71.8)		
District *					
Eastern	707 (29.0)	226 (32.0)	481 (68.0)	5.825	0.054
Western	722 (29.6)	209 (28.9)	513 (71.1)		
Middle	1011 (41.4)	269 (26.6)	742 (73.4)		
Marital Status					
Single	45 (1.8)	28 (62.2)	17 (37.8)	24.882	<0.001
Divorced/widowed	101 (4.1)	28 (27.7)	73 (72.3)		
Married	2294 (94.0)	648 (28.2)	1646 (71.8)		
Occupation					
Medical and health personnel	482 (19.8)	222 (46.1)	260 (53.9)	211.659	<0.001
Non-manual labor	841 (34.5)	318 (37.8)	523 (62.2)		
Manual labor	1117 (45.8)	164 (14.7)	953 (85.3)		
Smoking					
Never	2418 (99.1)	697 (28.8)	1721 (71.2)	2.088	0.352
Past (abstained ≥3 months)	11 (0.5)	2 (18.2)	9 (81.8)		
Current (over 6 months)	11 (0.5)	5 (45.5)	6 (54.5)		
Drinking					
Never	1636 (67.0)	420 (25.7)	1216 (74.3)	24.750	<0.001
Often (<3 times/week)	795 (32.6)	282 (35.5)	513 (64.5)		
Usually (3–7 times/week)	9 (0.4)	2 (22.2)	7 (77.8)		
Family cancer history					
No	2225 (91.2)	2225 (91.2)	1601 (72.0)	8.021	<0.05
Yes	215 (8.8)	215 (8.8)	135 (62.8)		
Education					
Second school or below	869 (35.6)	90 (10.4)	779 (89.6)	293.823	<0.001
Senior and vocational high school	437 (17.9)	104 (23.8)	333 (76.2)		
College or above	1134 (46.5)	510 (45.0)	624 (55.0)		
Education level of couple					
Second school or below	770 (31.6)	77 (10.0)	693 (90.0)	263.191	<0.001
Senior and vocational high school	522 (21.4)	125 (23.9)	397 (6.1)		
College or above	1148 (47.0)	502 (43.7)	646 (56.3)		
Per capita monthly household income					
<yuan 2000	829 (34.0)	142 (17.1)	687 (82.9)	125.623	<0.001
yuan 2000–3999	592 (24.3)	150 (25.3)	442 (74.7)		
≥yuan 4000	1019 (41.8)	412 (40.4)	607 (59.6)		
**Obstetric and gynecologic variables**					
Menstrual regularity					
No	546 (22.4)	110 (20.1)	436 (79.9)	25.971	<0.001
Yes	1894 (77.6)	594 (31.4)	1300 (68.6)		
Dysmenorrhea					
No	1711 (70.1)	487 (28.5)	1224 (71.5)	0.423	0.515
Yes	729 (29.9)	217 (29.8)	512 (70.2)		
Age of menarche					
<13	531 (21.8)	197 (37.1)	334 (62.9)	47.639	<0.001
13–15	1548 (63.4)	450 (29.1)	1098 (70.9)		
>15	361 (14.8)	57 (15.8)	304 (84.2)		
Leucorrhea					
No	1831 (75.0)	512 (28.0)	1319 (72.0)	2.828	0.093
Yes	609 (25.0)	192 (31.5)	417 (68.5)		
Couple’s circumcision					
Unknown	215 (8.8)	68 (31.6)	147 (68.4)	19.975	<0.001
No	1936 (79.3)	522 (27.0)	1414 (73.0)		
Yes	289 (11.8)	114 (39.4)	175 (60.6)		
Age of sexual debut					
≤20	665 (27.3)	167 (25.1)	498 (74.9)	6.228	<0.05
>20	1775 (72.7)	537 (30.3)	1238 (69.7)		
Number of sexual partners					
1	1976 (81.0)	534 (27.0)	1442 (73.0)	19.628	<0.001
2	344 (14.1)	119 (34.6)	225 (65.4)		
≥3	120 (4.9)	51 (42.5)	69 (57.5)		
Age of marriage					
≤20	299 (12.5)	43 (14.4)	256 (85.6)	32.274	<0.001
>20	2097 (87.5)	633 (30.2)	1464 (69.8)		
Frequency of sexual intercourse per month					
<4	1355 (55.5)	330 (24.4)	1025 (75.6)	30.036	<0.001
≥4	1085 (44.5)	374 (34.5)	711 (65.5)		
Number of pregnancies					
<2	534 (21.9)	224 (41.9)	310 (58.1)	57.106	<0.001
≥2	1906 (78.1)	480 (25.2)	1426 (74.8)		
Number of deliveries					
<2	929 (38.1)	305 (32.8)	624 (67.2)	11.568	<0.001
≥2	1511 (61.9)	399 (26.4)	1112 (73.6)		
Age at first parity					
≤20	170 (7.3)	24 (14.1)	146 (85.9)	17.036	<0.001
>20	2160 (92.7)	623 (28.8)	1537 (71.2)		
Menopausal status					
No	1901 (77.9)	687 (36.1)	1214 (63.9)	222.571	<0.001
Yes	539 (22.1)	17 (3.2)	522 (96.8)		
**Prior experience and medical history**					
Current contraceptive methods (like condom, contraceptives pills, sterilization, IUD)					
No	854 (35.0)	129 (15.1)	725 (84.9)	120.955	<0.001
Yes	1586 (65.0)	575 (36.3)	1011 (63.7)		
Ever had reproductive system disorders (like uterine myomas, cervical polyps, cervicitis, endometriosis)					
No	1722 (70.6)	488 (28.3)	1234 (71.7)	0.751	0.386
Yes	718 (29.4)	216 (30.1)	502 (69.9)		
Ever had uterine surgery (like hysterectomy with cervical preservation, cervical conization, cervical cerclage, polypectomy, LEEP)					
No	2118 (86.8)	611 (28.8)	1507 (71.2)	0.000	0.990
Yes	322 (13.2)	93 (28.9)	229 (71.1)		
Genital tract infections (like gonococcal, chlamydia trachomatis, mycoplasma, trichomoniasis)					
No	1952 (80.0)	544 (27.9)	1408 (72.1)	4.600	<0.05
Yes	488 (20.0)	160 (32.8)	328 (67.2)		
**HPV knowledge**					
I have ever heard of HPV					
No	684 (28.0)	50 (7.3)	634 (92.7)	214.868	<0.001
Yes	1756 (72.0)	654 (37.2)	1102 (62.8)		
I have ever heard of HPV-related diseases, such as genital warts and cervical cancer					
No	865 (35.5)	112 (12.9)	753 (87.1)	165.128	<0.001
Yes	1575 (64.5)	592 (37.6)	983 (62.4)		
Ever received HPV vaccination knowledge					
No	489 (20.0)	1 (0.2)	488 (99.8)	244.504	<0.001
Yes	1951 (80.0)	703 (36.0)	1248 (64.0)		
Ever received cervical screening knowledge					
No	947 (38.8)	184 (19.4)	763 (80.6)	66.940	<0.001
Yes	1493 (61.2)	520 (34.8)	973 (65.2)		
Regular screening is recommended following HPV vaccination					
No	191 (7.8)	23 (12.0)	168 (88.0)	120.602	<0.001
Unknown	526 (21.6)	72 (13.7)	454 (86.3)		
Yes	1723 (70.6)	609 (35.3)	1114 (64.7)		
I have ever participated in cervical cancer screening					
No	947 (38.8)	184 (19.4)	763 (80.6)	87.562	<0.001
National (free)	834 (34.2)	251 (30.1)	583 (69.9)		
Self-paid	659 (27.0)	269 (40.8)	390 (59.2)		
HPV state					
Negative	2093 (85.8)	610 (29.1)	1483 (70.9)	0.613	0.434
Positive	347(14.2)	94(27.1)	253(72.9)		

* District: eastern (Longgang, Pingshan, Dapeng), western (Nanshan, Baoan, Guangming), and central (Futian, Luohu, Yantian, Longhua).

**Table 4:** Univariate analyses of factors associated with intention to take HPV vaccination (N = 1736).

	Univariate Analysis				
	Intention to Take HPV Vaccination				
Demographic Variables	Overall	Yes (n = 1052)	No (n = 684)	c^2^	*p*
Age group					
30–39	645 (37.2)	489 (75.8)	156 (24.2)	114.779	<0.001
40–49	509 (29.3)	294 (57.8)	215 (42.2)		
50–59	479 (27.6)	223 (46.6)	256 (53.4)		
60–64	103 (5.9)	46 (44.7)	57 (55.3)		
Ethnicity					
Han	1652 (95.2)	1004 (60.8)	648 (39.2)	0.442	0.506
Other	84 (4.8)	48 (57.1)	36 (42.9)		
District *					
Eastern	481 (27.7)	270 (56.1)	211 (43.9)		
Western	513 (29.6)	332 (64.7)	181 (35.3)	7.663	<0.05
Middle	742 (42.7)	450 (60.6)	292 (39.4)		
Marital Status					
Single	17 (1.0)	11 (64.7)	6 (35.3)		
Divorced/widowed	73 (4.2)	38 (52.1)	35 (47.9)	2.430	0.297
Married	1646 (94.8)	1003 (60.9)	643 (39.1)		
Occupation					
Medical and health personnel	260 (15.0)	192 (73.8)	68 (26.2)		
Non-manual labor	523 (30.1)	377 (72.1)	146 (27.9)	87.256	
Manual labor	953 (54.9)	483 (50.7)	470 (49.3)		<0.001
Smoking					
Never	1721 (99.1)	1042 (60.5)	679 (39.5)		
Past (abstained ≥3 months)	9 (0.5)	6 (66.7)	3 (33.3)	0.233	0.890
Current (Over 6 months)	6 (0.3)	4 (66.7)	2 (33.3)		
Drinking					
Never	1216 (70.0)	709 (58.3)	507 (41.7)		
Often (<3 times/week)	513 (29.6)	340 (66.3)	173 (33.7)	10.527	<0.05
Usually (3–7 times/week)	7 (0.4)	3 (42.9)	4 (57.1)		
Family cancer history					
No	1601 (92.2)	960 (60.0)	641 (40.0)		
Yes	135 (7.8)	92 (68.1)	43 (31.9)	3.494	0.062
Education					
Second school or below	779 (44.9)	379 (48.7)	400 (51.3)	117.181	<0.001
Senior and vocational high school	333 (19.2)	193 (58.0)	140 (42.0)		
College or above	624 (35.9)	480 (76.9)	144 (23.1)		
Education level of couple					
Second school or below	693 (39.9)	348 (50.2)	345 (49.8)	89.647	<0.001
Senior and vocational high school	397 (22.9)	221 (55.7)	176 (44.3)		
College or above	646 (37.2)	483 (74.8)	163 (25.2)		
Per capita monthly household income					
<yuan 2000	687 (39.6)	350 (50.9)	337 (49.1)	60.918	<0.001
yuan 2000–3999	442 (25.5)	264 (59.7)	178 (40.3)		
≥yuan 4000	607 (35.0)	438 (72.2)	169 (27.8)		
**Obstetric and gynecologic variables**					
Menstrual regularity					
No	436 (25.1)	250 (57.3)	186 (42.7)	2.591	0.107
Yes	1300 (74.9)	802 (61.7)	498 (38.3)		
Dysmenorrhea					
No	1224 (70.5)	726 (59.3)	498 (40.7)	2.872	0.090
Yes	512 (29.2)	326 (63.7)	186 (36.3)		
Age of menarche					
<13	334 (19.2)	226 (67.7)	108 (32.3)	24.589	<0.001
13–15	1098 (63.2)	677 (61.7)	421 (38.3)		
>15	304 (17.5)	149 (49.0)	155 (51.0)		
Leucorrhea					
No	1319 (76.0)	779 (59.1)	540 (40.9)	5.448	<0.05
Yes	417 (24.0)	273 (65.5)	144 (34.5)		
Couple’s circumcision					
Unknown	147 (8.5)	87 (59.2)	60 (40.8)	3.830	0.147
No	1414 (81.5)	847 (59.9)	567 (40.1)		
Yes	175 (10.1)	118 (67.4)	57 (32.6)		
Age of sexual debut					
≤20	498 (28.7)	288 (57.8)	210 (42.2)	2.240	0.134
>20	1238 (71.3)	764 (61.7)	474 (38.3)		
Number of sexual partners					
1	1442 (83.1)	851 (59.0)	591 (41.0)	10.793	<0.05
2	225 (13.0)	149 (66.2)	76 (33.8)		
≥3	69 (4.0)	52 (75.4)	17 (24.6)		
Age of marriage					
≤20	256 (14.9)	125 (48.8)	131 (51.2)	17.218	<0.001
>20	1464 (85.1)	916 (62.6)	548 (37.4)		
Frequency of sexual intercourse per month					
<4	1025 (59.0)	589 (57.5)	436 (42.5)	10.306	<0.05
≥4	711 (41.0)	463 (65.1)	248 (34.9)		
Number of pregnancies					
<2	310 (17.9)	219 (70.6)	91 (29.4)	15.952	<0.001
≥2	1426 (82.1)	833 (58.4)	593 (41.6)		
Number of deliveries					
<2	624 (35.9)	419 (67.1)	205 (32.9)	17.495	<0.001
≥2	1112 (64.1)	633 (56.9)	479 (43.1)		
Age at first parity					
≤20	146 (8.7)	79 (54.1)	67 (45.9)	2.517	0.113
>20	1537 (91.3)	935 (60.8)	602 (39.2)		
Menopausal Status					
No	1214 (69.9)	806 (66.4)	408 (33.6)	56.746	<0.001
Yes	522 (30.1)	246 (47.1)	276 (52.9)		
Prior experience and medical history					
Current contraceptive methods (like condom, contraceptives pills, sterilization, IUD)					
No	725 (41.8)	388 (53.5)	337 (46.5)	26.149	<0.001
Yes	1011 (58.2)	664 (65.7%)	347 (34.3)		
Ever had reproductive system disorders (like uterine myomas, cervical polyps, cervicitis, endometriosis)					
No	1234 (71.1)	721 (58.4)	513 (41.6)	8.425	<0.05
Yes	502 (28.9)	331 (65.9)	171 (34.1)		
Ever had uterine surgery (like hysterectomy with cervical preservation, cervical conization, cervical cerclage, polypectomy, LEEP)					
No	1507 (86.8)	897 (59.5)	610 (40.5)	5.548	<0.05
Yes	229 (13.2)	155 (67.7)	74 (32.3)		
Genital tract infections (like gonococcal, chlamydia trachomatis, mycoplasma, trichomoniasis)					
No	1408 (81.1)	835 (59.3)	573 (40.7)	5.235	<0.05
Yes	328 (18.9)	217 (66.2)	111 (33.8)		
**HPV knowledge**					
I have ever heard of HPV					
No	634 (36.5)	268 (42.3)	366 (57.7)	140.509	<0.001
Yes	1102 (63.5)	784 (71.1)	318 (28.9)		
I have ever heard of HPV-related diseases, such as genital warts and cervical cancer					
No	753 (43.4)	337 (44.8)	416 (55.2)	139.827	<0.001
Yes	983 (56.6)	715 (72.7)	268 (27.3)		
Ever received HPV vaccination knowledge					
No	488 (28.1)	178 (36.5)	310 (63.5)	165.451	<0.001
Yes	1248 (71.9)	874 (70.0)	374 (30.0)		
Ever received cervical screening knowledge					
No	763 (44.0)	433 (56.7)	330 (43.3)	8.448	<0.05
Yes	973 (56.0)	619 (63.6)	354 (36.4)		
Regular screening is recommended following HPV vaccination					
No	168 (9.7)	48 (28.6)	120 (71.4)	137.674	<0.001
Unknown	454 (26.2)	224 (49.3)	230 (50.7)		
Yes	1114 (64.2)	780 (70.0)	334 (30.0)		
I have ever participated in cervical cancer screening					
No	763 (44.0)	433 (56.7)	330 (43.3)	24.462	<0.001
National (free)	583 (33.6)	341 (58.5)	242 (41.5)		
Self-paid	390 (22.5)	278 (71.3)	112 (28.7)		
HPV state					
Negative	1483 (85.4)	910 (61.4)	573 (38.6)	2.481	0.115
Positive	253 (14.6)	142 (56.1)	111 (43.9)		

* District: eastern (Longgang, Pingshan, Dapeng), western (Nanshan, Baoan, Guangming), and central (Futian, Luohu, Yantian, Longhua).

## Data Availability

The data presented in this study are available on request from the corresponding author.

## Supplementary Materials

The following supporting information can be downloaded at: https://www.mdpi.com/article/10.3390/vaccines13060561/s1, File S1. STROBE_checklist_cross-sectional. File S2. Questionnaire.
