# Supplementary material for: HPV Infection Prevalence, Vaccination-Related Knowledge, Attitudes, and Barriers Among Women Aged 30–64 in Shenzhen, China: A Cross-Sectional Study
Source: Vaccines (Basel). 2025 May 25;13(6):561. doi: 10.3390/vaccines13060561 (PMC12197789; doi:10.3390/vaccines13060561)
Supplement: Supplementary file 1 [file vaccines-13-00561-s001.zip › File S2.Questionnaire(Chinese).pdf]

问卷调查

第一部分：一般人口学资料

1.1 参与者 ID (匿名化) ___________________

1.2 您当前居住在哪个区: ___________________

1.3 您的民族:

□ 汉族

□ 其他民族 (请注明): _____

1.4 婚姻状况:

□ 未婚

□ 离婚/丧偶

□ 已婚

1.5 职业类型:

□ 医疗卫生行业人员

□ 脑力劳动人员

□ 体力劳动人员

1.6 吸烟史:

□ 否

□ 已戒烟 (至少 3 个月)

□ 仍在吸烟 (连续 6 个月以上)

1.7 饮酒史:

□ 从未

□ 偶尔 (<3 次/周)

□ 经常 (3-7 次/周)

1.8 受教育程度:

□ 初中及以下

□ 高中/中专

□ 大专及以上

1.9 您的配偶/性伴侣的受教育水平:

□ 初中及以下

□ 高中/中专

□ 大专及以上

1.10 您是否有肿瘤家族史:

□ 否

□ 是

1.11 您的配偶/性伴侣是否进行过包皮环切术？

□ 否

□ 是

□ 未知

1.12 您的家庭人均月收入: _____ (万元)

第二部分：妇产相关基本信息及既往病史

2.1 月经初潮年龄（周岁）: _____ 岁

2.2 您月经是否规律:

□ 否

□ 是

2.3 您是否痛经:

□ 否

□ 是

2.4 您的结婚年龄: _____ 岁 (如果未婚，跳过此题)

2.5 初次性行为年龄: _____ 岁

2.6 您有过几个性伴侣: _____ 个

2.7 （近一年）您平均每个月性生活频率: _____ 次

2.8 您是否存在白带异常:

□ 否

□ 是

2.9 是否绝经:

□ 否

□ 是 (绝经年龄: _____岁)

2.10 生育史: 孕: _____次，产_____次，初产年龄: _____ 岁(如果未生育，跳过此题)

2.11 目前使用的避孕措施:

□ 无

□有(比如: □ 避孕套 □ 避孕药 □ 绝育术 □ 宫内节育器 □ 其他_____)

2.12 您是否曾被医生诊断患有下列哪些生殖系统疾病？:

□ 无

□ 有 (比如: □ 宫颈炎 □ 子宫肌瘤 □ 宫颈息肉 □ 子宫腺肌病

□其他_____)

2.13 您曾经在医院进行过下列子宫病变手术吗？

□ 无

□有(比如: □ LEEP（宫颈环切术） □ 子宫全切术 (未切除宫颈) □ 息肉切除术 □ 宫腔镜手术 □ 其他_____)

2.14 您是否曾经患有下列生殖道感染相关疾病？

□ 无

□ 有 (比如: □ 念珠菌病感染 □ 梅毒螺旋体感染 □ 沙眼衣原体感染 □ 支原体感染 □ 其他_____)

第三部分: HPV 认识水平及接种意愿

3.1 我曾经听说过 HPV:

□ 否

□ 是

3.2 我曾经听说过 HPV 相关疾病（如宫颈癌、阴茎癌、肛门癌等）:

□ 否

□ 是

3.3 我曾经听说过 HPV 疫苗:

□ 否

□ 是

3.4 我有了解 HPV 疫苗的途径:

□ 否

□ 是 (比如: □ 传统媒体 □ 社交媒体 □ 家人、朋友交谈得知 □ 和医生、护士交谈得知 □ 社区、政府宣传)

3.5 您是否接种 HPV 疫苗:

□ 否

□ 是 → 跳转至 3.8

3.6 是否愿意自费接种 HPV 疫苗 (不考虑年龄因素):

□ 否

□ 是 → 跳转至 3.8

3.7 不愿意接种疫苗的原因 (多选):

□ 缺乏对 HPV 疫苗的了解

□ 认为自己没有患子宫颈癌的风险

□ 担心疫苗的有效性

□ 担心疫苗的安全性

□ 疫苗价格昂贵

□ 其他: _____

3.8 愿意接种疫苗的原因 (多选):

□ 可以预防宫颈癌

□ 担心自己感染 HPV

□ 伴侣受益

□ 其他: _____

3.9 HPV 疫苗接种后是否需要进行宫颈癌筛查:

□ 否

□ 是

□ 不知道

3.10 您最近一次参加宫颈癌筛查的是:

□ 从未参加过

□ 参加国家免费筛查

□ 参加自费筛查

3.11 我有了解 HPV 筛查的途径:

□ 否

□ 是 (比如: □ 传统媒体 □ 社交媒体 □ 家人、朋友交谈得知 □ 和医生、护士交谈得知 □ 社区、政府宣传)
