# Supplementary material for: HPV Infection Prevalence, Vaccination-Related Knowledge, Attitudes, and Barriers Among Women Aged 30–64 in Shenzhen, China: A Cross-Sectional Study
Source: Vaccines (Basel). 2025 May 25;13(6):561. doi: 10.3390/vaccines13060561 (PMC12197789; doi:10.3390/vaccines13060561)
Supplement: Supplementary file 1 [file vaccines-13-00561-s001.zip › File S2.Questionnaire.pdf]

Questionnaire

Section 1: Demographic Information

1.1 Participant ID (anonymous) __________________

1.2 Residential district in Shenzhen: __________________

1.3 Ethnicity:

□ Han

□ Other (specify): _____

1.4 Marital status:

□ Single

□ Divorced/Widowed

□ Married

1.5 Occupation type:

□ Healthcare worker

□ Non-manual labourer

□ Manual labourer

1.6 Smoking history:

□ Never

□ Former smoker (abstained =>3 months)

□ Current smoker (=> 6 months continuously)

1.7 Alcohol consumption:

□ Never

□ Often (<3 times/week)

□ Usually (3-7 times/week)

1.8 Education level:

□ Second school or below

□ Senior and vocational high school

□ College or above

1.9 Spouse/sexual partner’s education level:

□ Second school or below

□ Senior and vocational high school

□ College or above

1.10 Family history of malignancy:

□ No

□ Yes

1.11 Spouse/sexual partner’s circumcision status:

□ No

□ Yes

□ Unknown

1.12 Household monthly income per capita: _____ (10,000 CNY)

Section 2: Reproductive Health & Medical History

2.1 Age at menarche: _____ years

2.2 Regular menstrual cycles:

□ No

□ Yes

2.3 Dysmenorrhoea:

□ No

□ Yes

2.4 Age at marriage: _____ years (if unmarried, skip)

2.5 Age at first sexual intercourse: _____ years

2.6 Lifetime number of sexual partners: _____

2.7 Average monthly sexual frequency (past year): _____

2.8 Abnormal vaginal discharge:

□ No

□ Yes

2.9 Menopausal status:

□ No

□ Yes (age at menopause: _____ years)

2.10 Obstetric history:

Gravidity: _____

Parity: _____

Age at first delivery: _____ years(if not, skip)

2.11 Current contraceptive use:

□ None

□ Yes (specify: □ Condoms □ Oral contraceptives □ Sterilisation □ IUD □ Other_____)

2.12 Clinically diagnosed reproductive system disorders:

□ No

□ Yes (specify: □ Cervicitis □ Uterine fibroids □ Cervical dysplasia □ Adenomyosis □ Other_____)

2.13 Prior uterine procedures:

□ No

□ Yes (specify: □ LEEP □ Hysterectomy (cervix retained) □ Polypectomy □ Hysteroscopy □ Other_____)

2.14 History of reproductive tract infections:

□ No

□ Yes (specify: □ Candidiasis □ Treponema pallidum □ Chlamydia trachomatis □ Mycoplasma □ Other_____)

Section 3: HPV Awareness and Vaccination Intentions

3.1 I have ever heard of HPV:

□ No

□ Yes

3.2 I have ever heard of HPV-related diseases (e.g. genital warts, cervical/penile/anal cancer):

□ No

□ Yes

3.3 I have ever heard of HPV vaccines:

□ No

□ Yes

3.4 Sources of vaccine information (if aware):

□ No sources

□ Yes (specify: □ Traditional media □ Social media □ Family/friends □ Healthcare providers □ Community programmes/Government campaigns)

3.5 HPV vaccination status:

□ No → Proceed to 3.6

□ Yes → Skip to 3.8

3.6 Willingness to self-fund vaccination (if unvaccinated):

□ No → Proceed to 3.7

□ Yes → Skip to 3.8

3.7 Reasons for unwillingness (multiple selections allowed):

□ Lack of HPV vaccine knowledge

□ Perceived low cervical cancer risk

□ Concerns about vaccine efficacy

□ Concerns about vaccine safety

□ High cost

□ Other: _____

3.8 Motivations for willingness (multiple selections allowed):

□ Cervical cancer prevention

□ Personal HPV infection concerns

□ Partner benefits

□ Other: _____

3.9 Post-vaccination cervical cancer screening necessity:

□ No

□ Yes

□ Don’t know

3.10 Previous cervical cancer screening participation (most recent):

□ Never

□ National free screening programme

□ Self-funded screening

3.11 Sources of screening information:

□ No sources

□ Yes (specify: □ Traditional media □ Social media □ Family/friends □ Healthcare providers □ Community programmes/Government campaigns)
